# Milligan–Morgan hemorrhoidectomy, mucopexy, and hybrid combination for grade III hemorrhoids: a large retrospective cohort study

**DOI:** 10.1007/s10151-026-03291-y

**Published:** 2026-04-24

**Authors:** Gaetano Gallo, Veronica De Simone, Gianpiero Gravante, Salvatore Sorrenti, Simone Tierno, Federico Tomassini, Antonio Crucitti, Pierpaolo Sileri, Marco La Torre

**Affiliations:** 1 Colorectal Surgery Unit, IRCCS San Raffaele Scientific Institute, Vita-Salute University, Via Olgettina 60, 20132 Milan, Italy; 2 Department of General Surgery, Azienda Sanitaria Locale ASL Lecce, Ospedale “Francesco Ferrari”, Viale Francesco Ferrari 1, 73042 Casarano, Italy; 3 Department of Surgery, Sapienza University of Rome, Rome, Italy; 4 Department of Surgery, Ospedale Vannini, Rome, Italy; 5 Department of Surgery, Ospedale Grassi di Ostia, Rome, Italy; 6 Department of Surgery, Ospedale Cristo Re, Rome, Italy

**Keywords:** Mucopexy, Milligan-Morgan hemorrhoidectomy, combined approach, III degree Hemorrhoidal Disease, SurgicalApproach

## Abstract

**Background:**

Mucopexy has emerged as a nonexcisional alternative to Milligan–Morgan (MM) hemorrhoidectomy.

**Objective:**

This study compares MM, mucopexy, and hybrid MM/mucopexy procedures in patients with grade III hemorrhoidal disease (HD).

**Design:**

Retrospective cohort study.

**Setting:**

Tertiary referral setting.

**Patients:**

Symptomatic grade III HD treated between 2016 and 2018.

**Interventions:**

Four groups were defined: group 1 included patients treated with MM alone; group 2 received mucopexy on one pile combined with MM on the remaining two; group 3 underwent mucopexy on two piles with MM on the remaining one; and group 4 was treated with mucopexy alone.

**Main outcomes measures:**

Recurrence rates and patient-reported outcome measures (PROMs) using a visual-analogic scale (VAS) at 2 years, postoperative pain at 7 days, early complication rates at 30 days, and PROMs at 30 days.

**Results:**

A total of 686 consecutive patients with III-degree HD were included in the analysis. The most frequent approach was MM alone (group 1, *n* = 309, 45%), followed by mucopexy alone (group 4, *n* = 138, 20.2%), then the combined approaches (group 2, *n* = 120, 17.5%; group 3, *n* = 119, 17.3%). Group 4 had the lowest pain scores and the highest 30-day PROM (*p* < 0.001), whereas group 1 showed the best long-term results, with the lowest recurrence rate (4.2% versus 14.5% in group 4) and highest long-term PROM.

**Limitations:**

Retrospective analysis.

**Conclusions:**

Mucopexy and the hybrid procedures reduce morbidity and enhances early postoperative PROM in grade III HD. However, its higher recurrence rate suggests that a patient- and pile-specific approach may help balance efficacy with surgical invasiveness.

## Introduction

Hemorrhoidal disease (HD) is a common anorectal condition that frequently impairs quality of life, especially in its more advanced stages. For patients with grade III or IV HD who fail to respond to conservative treatments, surgery is often indicated. While several operative techniques have been developed, the choice between more traditional and newer approaches continues to be debated in the clinical practice [1].

Conventional hemorrhoidectomy, including open (Milligan–Morgan—MM) and closed (Ferguson) methods, remains the most widely performed surgical option owing to its proven long-term efficacy and low recurrence rates. Nonetheless, it is frequently associated with significant postoperative pain [2], prolonged healing times, and potential complications such as anal stenosis and minor continence disturbances. The adage *“no pain, no gain”* is often evoked to highlight the trade-off between the morbidity of excisional procedures and their superior durability in symptom control [3, 4]. Importantly, the severity of hemorrhoidal symptoms experienced by patients does not necessarily correlate with the anatomical grade of disease. Symptom burden is influenced by a multifactorial interplay, including psychological factors, which are not captured by the widely used Goligher classification—originally designed to guide surgical indication on the basis of the degree of prolapse. This classification system presents well-known limitations, particularly related to its interobserver variability [5, 6]. In light of these considerations, there is an increasing call to move beyond anatomy-based classifications toward a more patient-centered approach that prioritizes patient-reported outcomes and the real impact of symptoms on quality of life [7, 8].

In recent years, mucopexy—an anatomical repositioning of the prolapsed hemorrhoidal tissue through mucosal plication without excision or dearterialization—has gained attention as a nonexcisional alternative. Initially associated at the end of the transanal hemorrhoidal dearterialization [9–12], mucopexy has recently gained a dignity of his own as a standalone procedure for the treatment of HD [13, 14]. By avoiding extensive dissection and preserving anodermal structures, mucopexy may reduce postoperative discomfort, shorten recovery time, and lower the incidence of complications such as anal stenosis and delayed wound healing. Moreover, the absence of tissue excision makes the procedure repeatable in the case of recurrence.

This study aims to compare MM with mucopexy and a hybrid procedure (MM/mucopexy) according to the number and distribution of hemorrhoidal piles treated. The objective is to assess whether a selective use of mucopexy may provide a safer yet equally effective alternative to MM in patients with grade III HD.

## Materials and methods

### Study design

The study is a single-center retrospective study conducted at a high-volume tertiary referral center for proctological disorders and has been reported according to the Strengthening the Reporting of Observational Studies in Epidemiology (STROBE) guideline [15]. All patients treated between 2016 and 2018 for symptomatic Goligher grade III HD—defined as the presence of at least one pile requiring manual reduction—and who underwent either MM, mucopexy, or the hybrid procedure were retrospectively included and analyzed in this study [16]. Patients with other grades of severity were excluded: grades I and II because they received conservative or other nonexcisional treatments (lifestyle modifications, sclerotherapy, or rubber band ligation), and grade IV because they typically required extensive excisional surgery. In addition, patients who underwent procedures other than MM or mucopexy (e.g., Ferguson closed hemorrhoidectomy) were also excluded. A prospectively maintained database was used to select patients. Informed consent was obtained from all individual participants included in the study.

Diagnosis and severity of HD were established clinically by a single expert colorectal surgeon (M.L.T.), who also determined the indications and performed the surgical procedures. The preoperative assessment included a comprehensive proctological evaluation (including medical history, perineal examination, digital rectal examination, and anoscopy), as well as a colonoscopy and endoanal ultrasound to rule out any additional pathology. All patients were informed about the available surgical options—MM, mucopexy, or a hybrid combination of both—and provided informed consent accordingly. It was explained that the final choice of procedure would be made intraoperatively, on the basis of the findings of the examination under anesthesia.

The type of operation performed was evaluated and decided intraoperatively tailored to each single pile, given the clinical preoperative assessment could be misleading especially for II- and III-degree piles [5]. Patients were positioned in the lithotomy position, and antibiotic prophylaxis with cephalosporin was administered. The procedure was conducted under local anesthesia with a tailored anal block and mild sedation [17]. During anesthesia, the surgeon selected the appropriate treatment—mucopexy or MM—based on fibrotic consistency, elasticity, and volume of piles, with the aim of preserving tissue and minimizing invasiveness when characteristics appeared more favorable. For instance, a smaller, more elastic pile with minimal fibrotic changes was typically treated with mucopexy, as this approach allowed effective repositioning with reduced tissue trauma. Conversely, larger piles with significant fibrotic thickening and reduced mobility were more likely to be addressed with excision to ensure complete removal and reduce the risk of recurrence. Less symptomatic but structurally altered piles were also treated to prevent recurrence. Mucopexy was performed using a running 2/0 polyglactin 910 suture (Vicryl, Ethicon, Cincinnati, Ohio, USA). The stitch was initiated approximately 5 mm above the dentate line with an anchoring knot and continued proximally for 2–4 cm with 5–8 consecutive bites, depending on the length and volume of the hemorrhoidal pile, before securing the final knot. The continuous suture incorporated the mucosa and submucosa of the prolapsed hemorrhoidal cushions; by pushing the captured rectal mucosa proximally and applying gentle traction on the suture, the prolapsed tissue was lifted and repositioned. The procedure was carried out without the use of any dedicated or proprietary device, relying solely on standard surgical instruments and the polyglactin running suture. All procedures were conducted on a day surgery basis by a single well-trained surgeon (M.L.T.).

Four groups were defined according to the type of procedure performed: MM on all three piles without mucopexy (group 1), mucopexy on one pile and MM on the remaining two piles (group 2—hybrid procedure), mucopexy on two piles with MM on the remaining pile (group 3—hybrid procedure), and mucopexy on all piles without MM (group 4). MM was performed on a maximum of three piles to preserve elasticity, anal caliber and avoid long-term stenosis [18]. After surgery patients were encouraged to prevent passing hard stools and constipation by using laxatives (macrogol twice or three times a day) and a recommended oral dose of ketorolac tromethamine (10 mg every 6 h) on an as-needed basis, not exceeding 40 mg per day [19]. Moreover, they were advised to take regular warm sitz baths, to maintain a high-fiber diet, and to increase their fluid intake up to a minimum of 2 L of water daily.

Follow-up evaluations were conducted in person at 1 week, 1 month, 3 months, and 2 years postoperatively. Each visit included a comprehensive medical history and perianal examination, consisting of visual inspection, digital rectal examination, and anoscopy—with the latter two deferred at the 1-month follow-up. Presence of recurrence, incontinence and stenosis were defined according to the symptoms referred by the patients and direct clinical examinations performed by the operative surgeon. Recurrence was defined as the reappearance of symptomatic prolapse or bleeding requiring further medical or surgical intervention.

The aim of the study was to evaluate if the number of the mucopexies performed had an impact on postoperative results compared to MM. The primary outcomes were long-term recurrence rates and patient-reported outcome measures (PROMs) assessed using a 0–10 visual analog scale (VAS) at 2 years of follow-up. For the purpose of this study, PROMs reflected the patient’s global perception of the surgical result, mainly driven by postoperative pain and recurrence. Recurrence was defined as symptomatic prolapse or bleeding requiring medical or surgical intervention, based on the patient’s level of concern regarding the recurring symptoms and their impact on daily activities. Minor or occasional bleeding not requiring intervention was not considered a recurrence. Secondary outcomes included postoperative pain at 7 days (assessed using a 0–10 visual analogue scale -VAS), early complication rates evaluated at 30 days after surgery, and PROMs at 30 days (assessed using a 0–10 VAS). Postoperative complications were determined using the Clavien–Dindo classification [20]. The incidence of long-term recurrences, incontinence, stenosis were all assessed at 2 years of follow-up.

### Statistical analysis

All data were inserted into an Excel database (Microsoft, Redmond, Washington, USA) and analyzed with the Statistical Package for the Social Sciences Windows version 27.0 (SPSS, Chicago, Illinois, USA). Descriptive statistics used were the mean ± standard deviation for continuous parametric variables, and frequencies for categorical variables. Normality assumptions were demonstrated with histograms and the Shapiro–Wilk test. Analysis of comparison between groups was conducted with the analysis of variance (ANOVA) one-way test for continuous parametric variables, and chi-Square test for categorical variables (Fisher’s exact test if the counts in cells were inferior to 5). A *p* value less than 0.05 was considered statistically significant.

## Results:

During the study period, a total of 686 consecutive patients with III-degree HD were treated and included in the analysis. The mean age was 45.8 ± 15.2 years, 433 patients were males (63.1%) with no significant differences between groups (Table1). The most frequently performed procedure was hemorrhoidectomy alone (*n* = 309 patients, 45%; group 1), followed by mucopexy alone (*n* = 138 patients, 20.2%; group 4), single mucopexy combined with hemorrhoidectomy (*n* = 120, 17.5%; group 2), and double mucopexy combined with hemorrhoidectomy (*n* = 119, 17.3%; group 3).

**Table 1:** Demographic data, postoperative complications, early and late postoperative results divided by treatment groups

	All patients (*n* = 686)	Group 1 (*n* = 309)	Group 2 (*n* = 120)	Group 3 (*n* = 118)	Group 4 (*n* = 138)	*p*
Age (years)	46 ± 16	45 ± 17	45 ± 15	46 ± 19	47 ± 14	0.758
Sex (males)	433 (63.1%)	206 (66.7%)	79 (65.8%)	70 (58.8%)	78 (56.5%)	0.134
Postoperative bleeding	23 (3.4%)	14 (4.5%)	5 (4.2%)	2 (1.7%)	2 (1.4%)	0.264
Urinary retention	22 (3.2%)	10 (3.2%)	4 (3.3%)	4 (3.4%)	4 (2.9%)	1.000
Infections	3 (0.4%)	2 (0.6%)	0 (0%)	0 (0%)	1 (0.7%)	1.000
Overall postoperative early complications	48 (7.0%)	27 (8.7%)	9 (7.5%)	6 (5.0%)	6 (4.3%)	0.301
Reintervention	4 (0.6%)	4 (1.3%)	0 (0%)	0 (0%)	0 (0%)	0.438
Postoperative pain at 7 p.o. day (VAS)	4.9 ± 1.9	6.1 ± 1.5	4.9 ± 1.2	3.9 ± 1.3	3.1 ± 1.5	** < 0.001**
PROM at 30 days (VAS)	6.3 ± 1.2	5.9 ± 1.2	6.2 ± 0.9	6.0 ± 0.9	7.4 ± 1.1	** < 0.001**
Incontinence	8 (1.2%)	6 (1.9%)	2 (1.7%)	0 (0%)	0 (0%)	0.186
Stenosis	6 (0.9%)	4 (1.3%)	2 (1.7%)	0 (0%)	0 (0%)	0.351
Recurrence	54 (7.9%)	13 (4.2%)	8 (6.7%)	13 (10.9%)	20 (14.5%)	**0.001**
PROM at 2 years (VAS)	6.7 ± 1.3	7.8 ± 1.0	6.1 ± 1.0	6.0 ± 0.9	5.6 ± 0.9	** < 0.001**

*VAS* Visual Analogic Scale (0–10)

Postoperative complications were observed in 48 patients, accounting for 7% of the total cohort. These included 23 cases of bleeding (3.4%), 3 cases of wound infection (0.4%), and 22 cases of urinary retention requiring catheterization (3.2%). No statistically significant differences in complication rates were found among the groups (Table 1). Focusing on postoperative bleeding alone (23 out of 686 patients, 3.4%), the highest incidence was recorded after hemorrhoidectomy (4.5%, 14 out of 309), while the lowest occurred following mucopexy alone (1.4%, 2 out of 138). However, these differences were not statistically significant. Reoperation was required in four cases (0.6%), all due to postoperative bleeding in group 1. Six cases of anal stenosis were reported (1%), with four in group 1 (1.3%) and 2 in group 2 (1.7%; *p* = 0.3); one of these patients required anoplasty, while the others responded to conservative treatment. In addition, anal incontinence occurred in eight patients (1.2%), of whom six were in group 1 (1.9%) and two in group 2 (1.7%; *p* = 0.2). All cases of incontinence were managed successfully with pelvic floor rehabilitation.

### Group comparisons

**Fig. 1:**
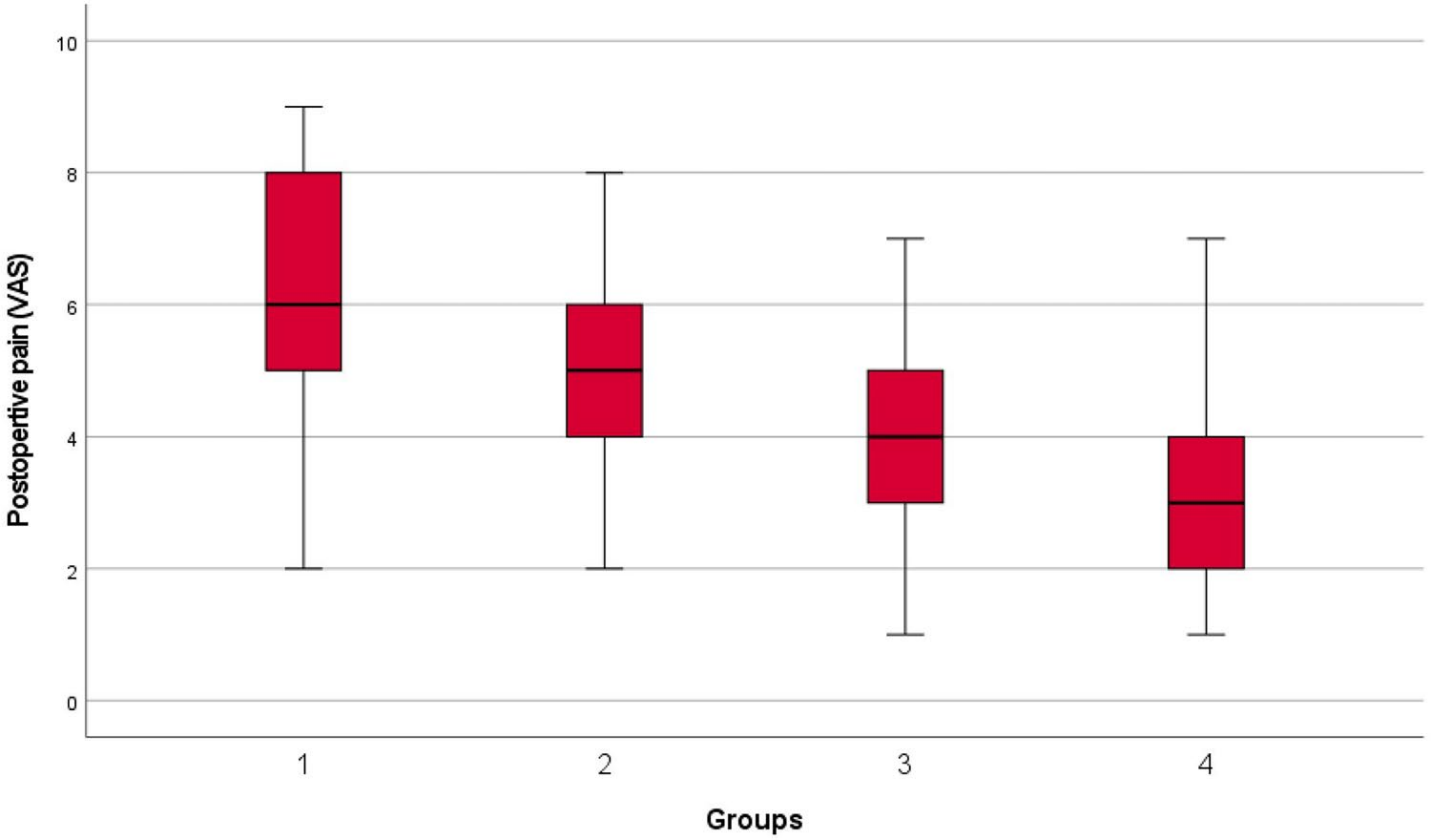
Box plot graphs comparing pain on the 7th postoperative day among groups. *VAS* Visual Analogic Scale (0–10)

**Fig. 2:**
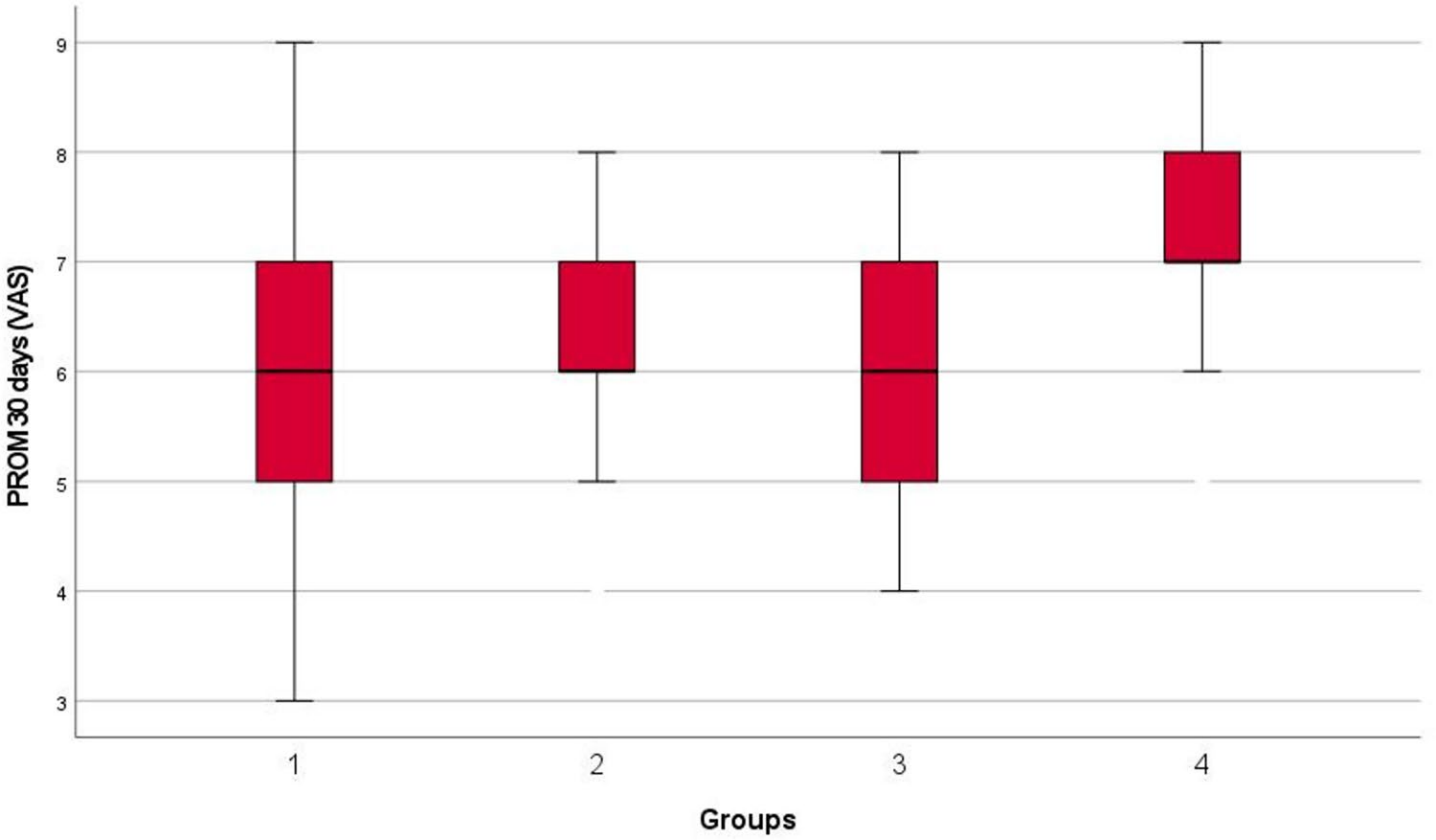
Box plot graphs comparing patient-reported outcome measure (PROM) assessed with the Visual Analogic Scale (0–10) at the 30th postoperative day among groups

**Fig. 3:**
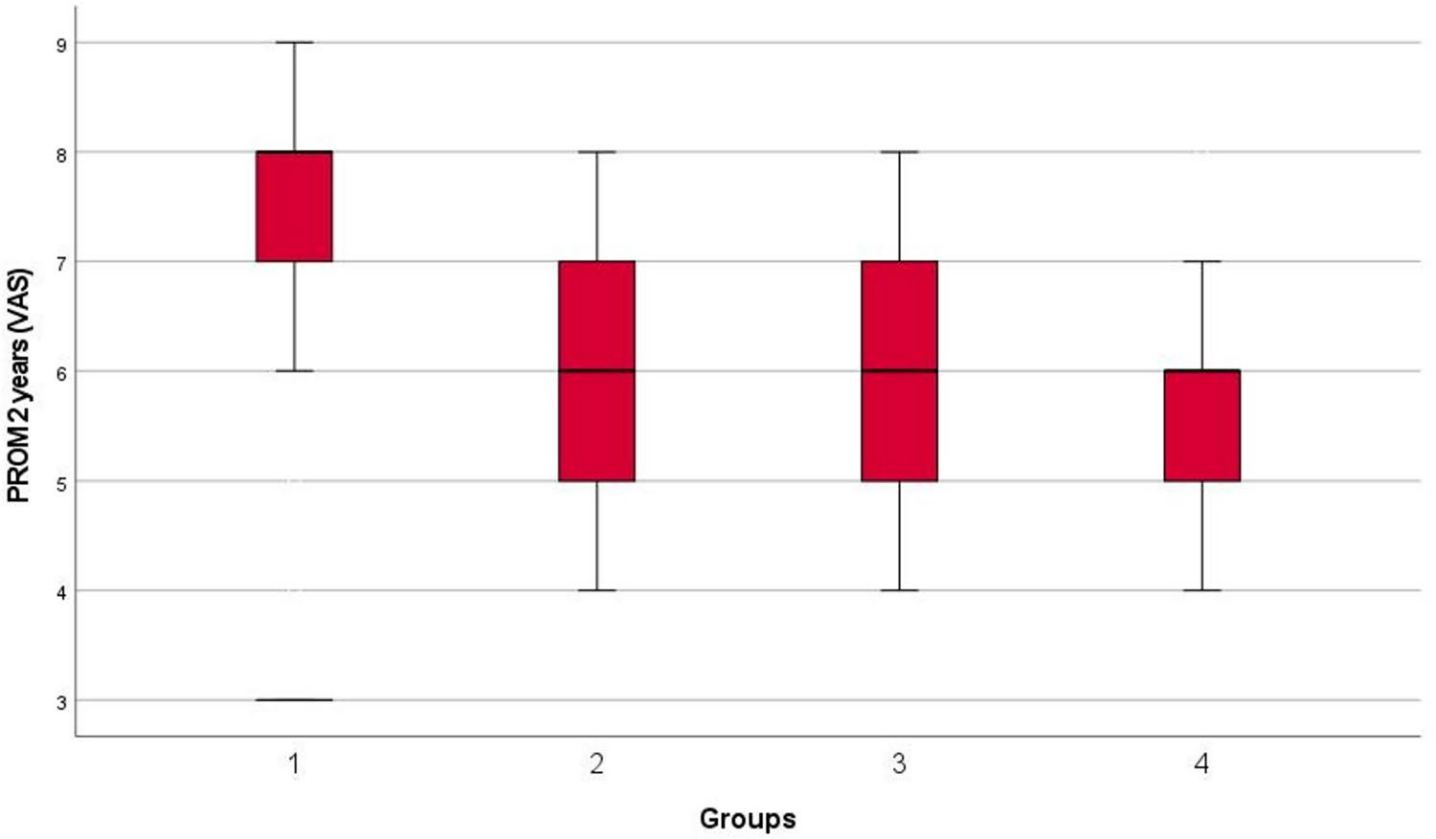
Box plot graphs comparing patient-reported outcome measure (PROM) assessed with the Visual Analogic Scale (0–10) at the 2nd postoperative year among groups

A one-way ANOVA with Bonferroni post-hoc multiple comparisons was performed to evaluate differences among the four intervention groups (groups 1, 2, 3, and 4) for the following parameters: pain at 7 days (VAS 0–10), PROM at 30 days (VAS 0–10), and PROM at 2 years (VAS 0–10). Statistically significant differences were observed among all four groups for pain at 7 days postop (*p* < 0.001 for all pairwise comparisons; Table 2; Fig. 1). Group 4 reported the lowest mean pain score, indicating a better postoperative pain profile. Significant differences were found between most groups for PROM at 30 days (*p* < 0.001), except between group 2 and group 3, where no statistically significant difference was observed (*p* = 0.058; Table 2; Fig. 2). Group 4 showed the highest PROM scores at this time point. Statistically significant differences were again observed between most groups for PROM at 2 years (*p* < 0.001), except between group 2 and group 3 (*p* = 1.000; Table 2; Fig. 3). However, in contrast to the 30-day results, group 1 reported the highest long-term PROM, while group 4 had the lowest PROM score at 2 years. While group 4 achieved the best results in terms of short-term pain and early PROM, long-term PROM appeared to decline compared with the other groups 1, 2, and 3.

**Table 2:** Statistical significance (*p*-values) for pairwise comparisons between groups for each of the three variables: pain at 7 days, patient-reported outcome measure (PROM) at 30 days, and PROM at 2 years

Group comparison	Pain 7 days (VAS)	PROM 30 days (VAS)	PROM 2 years (VAS)
1 versus 2	*p* < 0.001	*p* < 0.001	*p* < 0.001
1 versus 3	*p* < 0.001	*p* < 0.001	*p* < 0.001
1 versus 4	*p* < 0.001	*p* < 0.001	*p* < 0.001
2 versus 3	*p* < 0.001	*p*** = 0.058**	***p* = 1.000**
2 versus 4	*p* < 0.001	*p* < 0.001	*p* < 0.001
3 versus 4	*p* < 0.001	*p* < 0.001	*p* = 0.003

*VAS* Visual Analogic Scale (0–10)

## Discussions

In recent years, hybrid approaches to the treatment of hemorrhoids have become increasingly common in clinical practice, particularly for grade III and IV disease. These techniques combine elements of both traditional and minimally invasive procedures, aiming to maximize effectiveness while minimizing postoperative pain, complications, and recovery time [14, 21, 22]. In our study, groups 2 and 3 (hybrid procedures) represent a middle-ground approach, combining excisional and nonexcisional strategies tailored to the intraoperative characteristics of each pile. By performing MM on the more fibrotic or prolapsed piles and mucopexy on the less severe ones, this approach aims to reduce overall postoperative morbidity compared with MM. It is also important to note that these techniques address different anatomical problems and are not intended as interchangeable solutions for the same degree of disease. For this reason, the purpose of the study is not merely to compare the techniques in isolation, but to demonstrate the clinical rationale for using a combination of approaches when the volume and the heterogeneity of the disease makes uniform treatment inappropriate. This concept supports the growing paradigm shift toward “tailored surgery” in the management of HD, which aims to optimize treatment on a pile-specific, patient-specific basis rather than relying on uniform approaches for all patients [14, 22].

In fact, the three surgical strategies have complementary roles: mucopexy, whether performed alone or selectively on less symptomatic piles, demonstrates clear advantages in terms of reduced postoperative pain and improved short-term PROM. No additional costs were incurred beyond those related to the suture material itself, as the procedure does not require any dedicated device (such as Doppler-guided Transanal Hemorrhoidal Dearterialization—THD), which contributes to its practicality and affordability. Conversely, MM, despite being associated with greater postoperative discomfort, remains superior in terms of long-term outcomes and lower recurrence rates. Positioned between these two, the hybrid approaches integrate the benefits of both techniques, tailoring the treatment to the intraoperative assessment of pile severity. Drawing on a large and homogeneous cohort of patients with grade III HD treated with MM, mucopexy, or combinations, this study provides a robust analysis of the incremental value of mucopexy relative to the number of piles addressed. To date, it represents the largest dataset available for this specific subgroup.

The findings underscore the importance of a tailored surgical approach that balances invasiveness with efficacy, supporting individualized treatment plans on the basis of each patient’s anatomical and clinical profile. Tailoring the surgical approach to the individual patient symptoms and wishes, and to the specific intraoperative characteristics of each hemorrhoidal pile, may offer a more balanced alternative between invasiveness and long-term efficacy. While mucopexy showed a clear benefit in terms of early postoperative pain and short-term PROM, its higher long-term recurrence rate indicates that this technique should not yet be considered a full substitute for excisional surgery in all cases. However, when selectively applied—particularly for less prolapsed or symptomatic piles—it may enhance overall outcomes by reducing morbidity without significantly compromising efficacy. Notably, considering the particularly long follow-up period of 5 years, the observed recurrence rate of 14.5% does not appear especially unfavorable for a nonexcisional technique that offers a more comfortable postoperative course compared with MM, and is consistent with the results reported by Pucher et al. (17.5%) and Giordano et al. (10.5%) in their respective meta-analyses [23, 24]. The absence of Doppler-guided selective hemorrhoidal artery ligation does not seem to compromise outcomes, as recently demonstrated by a systematic review and meta-analysis [25].

Our findings also offer valuable data to support a more informed consent process, enabling patients to actively participate in the decision-making. Patients can express whether they prefer a procedure associated with greater postoperative pain but a lower risk of recurrence, or one with less immediate discomfort but a higher chance of recurrence and potential need for further surgical intervention. This concept supports the growing paradigm shift toward “tailored surgery” in the management of HD, which aims to optimize treatment on a piles-specific, but also patient-specific basis rather than relying on a uniform approach for all patients [14]. From this perspective, the ideal candidate for MM would be a patient who prioritizes long-term PROM and low recurrence rates, and who intraoperatively presents with large hemorrhoidal piles characterized by significant fibrotic thickening and reduced mobility. Conversely, mucopexy may be more appropriate for patients seeking a faster recovery and less postoperative pain, particularly in the presence of smaller, more elastic piles with minimal fibrotic changes. Hybrid procedures offer an intermediate solution, combining the advantages of both techniques based on intraoperative assessment and individual patient priorities. Further research incorporating validated PROMs and longer follow-up is warranted to refine this strategy and better identify the ideal candidates for limited or selective mucopexy [8].

This study presents several limitations. First, the retrospective nature of the study involves recall bias, nonrandom allocation, and surgeon-driven selection bias that all have to be taken into account. In particular, because the choice between mucopexy and MM was made intraoperatively by the operating surgeon on the basis of real-time evaluation of pile consistency, volume, and fibrosis, the groups inherently reflect different underlying anatomical severities. This intrinsic imbalance is unavoidable in retrospective analyses of tailored surgical approaches and must be considered when interpreting the comparative results. Second, it is a single-center analysis conducted at a tertiary referral institution with high specialization in colorectal surgery. While this setting ensures standardized surgical procedures and consistent diagnostic protocols, it may limit the generalizability of the findings to centers with different levels of expertise or resource availability. Third, no closed (Ferguson) hemorrhoidectomy procedures were included, as MM was the most frequently adopted surgical approach. While this allowed for a direct comparison with mucopexy, it excluded a potentially less painful alternative that could yield different outcomes; this limitation should be addressed in future dedicated studies. Similarly, although mucopexy as a standalone procedure is cost-effective, it can also be combined with Doppler-guided techniques such as THD [26, 27], or with laser-assisted approaches (HeLPexx) [28, 29]. It would be of interest to evaluate how these combinations might affect recurrence rates, long-term PROM, and associated costs. Finally, while outcomes were assessed through clinical recurrence and a VAS-based PROM, we acknowledge that a single VAS score cannot capture all dimensions of patient satisfaction. In this study, the PROM reflects a self-reported global appraisal of the surgical result, largely driven by postoperative symptoms and recurrence, and should not be interpreted as a comprehensive measure of overall patient satisfaction, which encompasses broader domains. Future studies should incorporate validated, multidimensional PROMs to more fully characterize patient-centered outcomes and strengthen these findings.

## Conclusions

MM remains the technique associated with the lowest 2-year recurrence rates. Mucopexy—performed alone or within a hybrid approach—provides clear advantages in terms of reduced postoperative pain. The hybrid strategies, which combine excision of the more fibrotic piles with mucopexy of the less severe ones, offer an intermediate balance between invasiveness and long-term durability. Overall, these findings support a tailored, patient- and pile-specific approach that aims to minimize unnecessary excision while maintaining effective long-term control of symptoms.

## Data Availability

Data is provided upon reasonable request by the Authors.
